# DDR1 promotes LoVo cell proliferation by regulatingenergy metabolism: Effect of DDR1 on CRC cell metabolic reprogramming

**DOI:** 10.3724/abbs.2022038

**Published:** 2022-05-18

**Authors:** Bin Xiong, Zehui Xie, Feixue Song, Huiling Chen, Xiaojuan Wang, Zhengxu Jin, Tiyun Han, Yi Li, Dekui Zhang

**Affiliations:** 1 Department of Oncology The Second Hospital of Lanzhou University Lanzhou 730030 China; 2 The Second Clinical Medical College Lanzhou University Lanzhou 730030 China; 3 Laboratory of Digestive Disease The Second Hospital of Lanzhou University Lanzhou 730030 China; 4 School/Hospital of Stomatology Lanzhou University Lanzhou 730000 China; 5 Department of Gastroenterology The Second Hospital of Lanzhou University Lanzhou 730030 China

**Keywords:** colorectal cancer, DDR1a, proliferation, lactic acid, Warburg effect

## Abstract

Cellular energy metabolism dysregulation is associated with colorectal cancer (CRC) development and progression. Discoidin domain receptor 1a (DDR1a), one of the five DDR1 isoforms, is closely related to cell proliferation, invasion, and apoptosis in various tumors. Whether it participates in cellular metabolic reprogramming and regulates CRC initiation and progression remains unclear. In this study, we compared the expression of DDR1 in CRC tissues and adjacent tissues from 126 postoperative CRC samples. Moreover, lentivirus-mediated DDR1a overexpression and knockdown were performed in LoVo cells, and cell viability and proliferation were determined by CCK-8 and BrdU assays, respectively. Oxygen consumption rate, extracellular acidification rate, and lactate production were used to determine the effect of DDR1a on metabolic reprogramming. Clinically, CRC patients with high DDR1 expression had poor differentiation and were at an advanced TNM stage. DDR1a promoted LoVo cell proliferation, mitochondrial function, and extracellular acidification. Moreover, DDR1a knockdown inhibited intracellular lactic acid production in LoVo cells, while a pyruvate kinase inhibitor (diamide, 200 μM) significantly reversed this progression. Taken together, our results reveal that DDR1 plays a crucial role in maintaining intracellular environment homeostasis through metabolic reprogramming.

## Introduction

Colorectal cancer (CRC) is the third most common cancer worldwide, with approximately 1.8 million new cases diagnosed in 2018 [1]. Recent studies have reported that some molecular biomarkers can be used to diagnose and monitor CRC in its early stages [ 2, 3]. Accordingly, the overall survival of patients with CRC has improved. However, due to chemotherapy resistance, most patients with advanced CRC do not benefit from an initial fluorouracil-based regimen and new biotherapy [4]. Furthermore, recurrent chemotherapy-resistant CRC is incurable; thus, CRC remains a life-threatening disease. As such, there is an urgent need to develop novel therapeutic strategies for CRC treatment, and cell biology may provide new insights into solving this problem [5].

The energy metabolism reprogram, an important hallmark of cancer, can facilitate cancer progression [ 6– 8]. CRC cells are usually rewired for glycolysis, producing lactic acid, even under aerobic conditions (the Warburg effect). Thus, CRC tumors are found in an acidic microenvironment [9]. Under such conditions, CRC cells exhibit higher invasion and migration capability, resulting in chemotherapy resistance [ 10– 13]. Some genes and biosynthetic pathways, such as KRAS [14], TP53 [15], and Wnt signaling [16], are involved in metabolic reprogramming. However, it is regrettable that the metabolic pathways that significantly influence CRC initiation and progression have not yet been confirmed. Therefore, further studies on energy metabolism remodeling in CRC are urgently required.

Discoidin domain receptors (DDRs) were initially discovered in 1997 via homology cloning based on their catalytic kinase domains [ 17, 18]. DDR1a is one of five DDR1 isoforms that are generated by alternative splicing [19]. Previous studies have shown that DDR1 is highly expressed in many cancers, including gastric [20], lung [21], hepatocellular cancers [22], glioma [23], and prostate cancer [24]. These key findings implicate DDR1 as a major contributor to tumor initiation, growth, and metastasis [ 25, 26]. Nonetheless, the role of DDR1 in CRC and the underlying mechanism in the regulation of cellular metabolism are still unclear.

In this study, we compared the differences in DDR1 expression between CRC tissues and adjacent tissues, and evaluated the correlation between DDR1 expression and clinicopathological characteristics. Furthermore, to study the function of DDR1 and its effect on glycolysis and oxidative phosphorylation, we analyzed the oxygen consumption rate (OCR) and extracellular acidification rate (ECAR) in LoVo cells after lentivirus-mediated DDR1a overexpression and knockdown. In summary, this study aimed to investigate the effect of DDR1 on growth and energy metabolism in CRC, as well as its underlying mechanism, and to provide novel insights into its possible use as a therapeutic target.

## Materials and Methods

### Patients and tissue specimens

This study followed the national guidelines and protocols of the National Institutes of Health and was approved by the local Ethics Committee for the Care and Use of Human Tissue and Pathological Specimens of the Second Hospital of Lanzhou University (Lanzhou, China). One hundred and twenty-six patients, who were newly diagnosed with CRC and underwent surgical resection without any preoperative therapy at the Second Hospital of Lanzhou University from January 2018 to August 2018, were selected as study participants. Written informed consent was obtained from all patients before resection. The pathological T/N/M statuses and cancer stages of the selected patients were defined according to the staging system of the American Joint Committee on Cancer (AJCC) 8th edition [27]. All specimens were reviewed by two pathologists to confirm the diagnosis. The clinical characteristics of the CRC patients are shown in Table 1.

**Table:** **Table1** Correlation of DDR1 expression with clinicopathological features in CRC

Clinicopathological parameter	Cases ( *n*)	DDR1 expression ( *n*, %)	*P* value
Negative	Positive	Strong positive
Age (years)	
>60	74	16 (21.6)	24 (32.4)	34 (46.0)	*P*=0.946
≤60	52	14 (27.0)	19 (36.5)	19 (36.5)
Gender	
Male	72	14 (19.4)	25 (34.7)	33 (45.9)	*P*=0.381
Female	54	16 (29.7)	18 (33.3)	20 (37.0)
Greatest tumor diameter (cm)	
>4	60	7 (11.7)	24 (40.0)	29 (48.3)	*P*<0.05
≤4	66	23 (34.8)	19 (28.8)	24 (36.4)
Differentiation	
High	17	12 (70.6)	5 (29.4)	0 (0.0)	*P*<0.001
Moderate	75	18 (24.0)	33 (44.0)	24 (32.0)
Poor, other	34	0 (0.0)	5 (14.7)	29 (85.3)
pT	
T1	11	11 (100.0)	0 (0.0)	0 (0.0)	*P*<0.001
T2	11	8 (72.7)	3 (27.3)	0 (0.0)
T3	32	8 (25.0)	15 (46.9)	9 (28.1)
T4	72	3 (4.2)	26 (36.1)	43 (59.7)
pN	
N0	76	28 (36.8)	28 (36.8)	20 (26.4)	*P*<0.001
N1-2	50	3 (6.0)	14 (28.0)	33 (66.0)
AJCC stage	
I	18	17 (94.4)	1 (5.6)	0 (0.0)	*P*<0.001
II	56	11 (19.6)	26 (46.4)	19 (34.0)
III	27	2 (7.4)	12 (44.4)	13 (48.2)
IV	25	0 (0.0)	4 (16.0)	21 (84.0)
Vascular invasion	
Negative	28	24 (85.7)	3 (10.7)	1 (3.6)	*P*<0.001
Positive	98	6 (6.1)	40 (40.8)	52 (53.1)

AJCC, American Joint Committee on Cancer; CRC, colorectal cancer.

The *χ* ^2^ test or Fisher’s exact test was used to analyze the association between DDR1 expression and clinicopathological features.

### Immunohistochemistry analysis

Immunohistochemistry analyses were performed using standard protocols. Briefly, the slides were incubated with anti-DDR1 antibody (D1G6 XP ^®^ Rabbit mAb 5583; Cell Signaling Technology, Danvers, USA) overnight at 4°C. The slides were then incubated with an HRP-conjugated anti-rabbit secondary antibody (Roche, Basel, Switzerland). Finally, the slides were stained with 3-diaminobenzidine and subsequently observed and photographed under an inverted microscope (IX71; Olympus, Tokyo, Japan). DDR1 expression was evaluated by two pathologists who were blinded to the clinicopathological features of the patients. The immunohistochemical scores of DDR1 expression are shown in Table 2.

**Table:** **Table2** Immunohistochemical scores of DDR1 expression in CRC

	DDR1 positive cells percentage
≤5%	5%–30%	30%–60%	≥60%
Scores	0	1	2	3

The proportion of cells with DDR1 positive staining was found to be between 0 and 100%. The expression of DDR1 was determined as positive when both sites received a score of 2 or 3. The intensity scores were then added to obtain a total score ranging from 0 to 6. Cases with scores of 0–2 were considered negative, 3–4 positive, and 5–6 strongly positive.

### Cell culture and treatment

The human colorectal carcinoma cell line LoVo, kindly provided by Prof. You-Cheng Zhang of the Second Department of General Surgery, the Second Hospital of Lanzhou University, was maintained in ATCC-formulated Ham’s F12K medium supplemented with 10% fetal bovine serum (Thermo Fisher Scientific, Waltham, USA), 100 U/mL penicillin, and 100 μg/mL streptomycin in a humidified atmosphere containing 5% CO _2_ at 37°C. For diamide (a pyruvate kinase inhibitor) treatment, LoVo cells were incubated with different diamide concentrations (0, 50, 100, 200, and 400 μM) in Ham’s F12K medium for 15 min.

### DDR1a knockdown/overexpression LoVo cells

To verify the effect of DDR1a on cell proliferation and energy metabolism in LoVo cells, shRNA-mediated DDR1a knockdown (shDDR1a) and DDR1a overexpression (DDR1a ^high^) were performed using materials from GenePharma (Shanghai, China). Two vectors were used to target the shDDR1a: LV3-DDR1a-homo-1733 (shDDR1a-1) and LV3-DDR1a-homo-1043 (shDDR1a-2) with sequences 5′-GGGACACTATCCTCATCAACA-3′ and 5′-GGCTGGATGACTTTAGGAAGA-3′, respectively. The sequence of the negative control LV3-DDR1a shRNA (LV3) was 5′-TCTCCGAACGTGTCACGT-3′. The vector was selected to construct shDDR1a based on qPCR and western blot analysis. Next, LoVo cells were transfected with lentivirus-mediated DDR1a vectors carrying green fluorescent protein (GFP) and an anti-puromycin gene. Thereafter, LoVo cells were infected with recombinant lentiviruses for 72 h using medium containing 1.2 g/mL puromycin to screen positive transfected cell lines in each group, with the involvement of their control vectors (DDR1a ^high^-NC and shDDR1a-NC).

### Cell viability and proliferation assay

The cell viability and proliferation of LoVo cells were assessed using a CCK-8 assay kit (Dojindo Molecular Technologies Inc., Kumamoto, Japan) and BrdU cell proliferation ELISA kit (ab126556; Abcam, Cambridge, UK), respectively. Transfected LoVo cells were seeded into 96-well plates at a density of 5000 cells/well (for the CCK-8 assay) or 1×10 ^4^ cells/well (for the BrdU assay) and incubated for 0, 24, 48, and 72 h. For the CCK-8 assay, 10 μL of CCK-8 solution was added to each well, and the cells were incubated for 2 h according to the manufacturer’s instructions. For the BrdU assay, BrdU was added to each well and incubated for 24 h. After incubation, the cells were fixed in fixing solution (200 μL) for 30 min. Subsequently, the cells were removed from the fixing solution and incubated with 100 μL anti-BrdU antibody for 2 h at room temperature. Next, 100 μL peroxidase-conjugated goat anti-mouse IgG was added to the cells and incubated for 30 min at room temperature. The absorbance was measured at 450 nm with an automatic spectrophotometer (PowerWave X; Bio-Tek, Winooski, USA).

### Mitochondrial staining

Mitochondrial activity was evaluated using MitoTracker Orange (CMTMRos, M7510; Invitrogen, Waltham, USA). Briefly, the transfected cells were incubated with pre-warmed MitoTracker solution (diluted in serum-free Ham’s F12 medium to a final concentration of 20 nM) for 20 min at 37°C in the dark. The cells were then washed thrice with PBS and fixed with 3.7% paraformaldehyde for 30 min at room temperature. Finally, the cells were observed under a fluorescence microscope (BX53F; Olympus). Fluorescence intensity was determined using ImageJ software. Cells were counted in five random fields per slide, and at least three slides were counted.

### Extracellular acidification rate and oxygen consumption rate measurement

Extracellular acidification rate (ECAR) and oxygen consumption rate (OCR) were measured using a Seahorse XF-24 Extracellular Flux Analyzer (Seahorse Bioscience, North Billerica, USA). Briefly, 8×10 ^4^ cells/well were seeded in XF24 cell culture microplates (Seahorse Bioscience) with 250 μL of Ham’s F12K medium and incubated overnight. To determine ECAR, the cells were plated in XF Seahorse medium with 2 mM glutamine using the following concentrations of injected compounds: 1 μM oligomycin, 50 mM 2-DG, and 10 mM glucose. For OCR determination, the cells were plated in XF Seahorse medium with 25 mM glucose, 2 mM glutamine, and 1 mM sodium pyruvate in the mitochondrial stress test using the following concentrations of injected compounds: 1 μM oligomycin, 250 nM carbonyl cyanide 4-(trifluoromethoxy)phenylhydrazone (FCCP), and 1 mM rotenone+1 mM antimycin A (Sigma-Aldrich, St Louis, USA).

### Intracellular lactate content assay

The intracellular lactate content of the cells was determined using a lactic acid test kit (Nanjing Jiancheng Bioengineering Institute, Nanjing, China). The IC _50_ concentrations of diamide were primarily determined by CCK8 assay, and 200 μM was selected to evaluate the effect of DDR1a-mediated pyruvate kinase (PK) activation on intracellular lactate content changes. Cells were seeded in 96-well plates at a density 1×10 ^4^ cells/well, cultured in complete medium or treated with 200 μM diamide for 15 min (blank control group and zero adjustment wells were also set), and the OD value was read at 530 nm using the automatic spectrophotometer (PowerWave X; Bio-Tek). Three independent experiments were conducted.

### Colorimetric phosphofructokinase, hexokinase, and pyruvate kinase activity assay

Phosphofructokinase (PFK), hexokinase (HK), and PK activities were determined using PFK, HK, and PK activity colorimetric assay kits (Nanjing Jiancheng Bioengineering Institute). First, the cells were seeded into 96-well plates at a density of 1×10 ^4^ cells/well and cultured for 48 h. Thereafter, the supernatant medium was removed and incubated with cell lysis buffer on ice for 10 min. Next, the lysates were centrifuged at 10,000 *g* for 2 min, and a reaction buffer was added to each well and incubated for 10 min at 37°C. Finally, the absorbance was read at 340 nm using the automatic spectrophotometer. Meanwhile, the protein concentration of the extracts was determined by the Bradford assay.

### qRT-PCR

The transfected LoVo cells were incubated for 48 h, after which they were seeded into 6‐well plates (2×10 ^5^ cells per well). Then RNA was extracted using the TRIzol reagent (Invitrogen, Carlsbad, USA) and was detected using a reverse transcription kit and an amplification kit (TaKaRa, Dalian, China) following the manufacturer’s instructions. The sequences of the primers were as follows: *DDR1a* forward, 5′-ATGGAGCAACCACAGCTTCTC-3′, reverse, 5′-CTCAGCCGGTCAAACTCAAACT-3′; *β-actin* forward, 5′-CTCCATCCTGGCCTCGCTGT-3′, reverse, 5′-GCTGTCACCTTCACCGTTCC-3′. Data were normalized to the *β-actin* expression and calculated by the 2 ^−ΔΔCt^ method.

### Western blot analysis

The transfected LoVo cells, untreated or treated with diamide, were washed three times with ice-cold PBS, and proteins were extracted using RIPA lysis buffer containing 1 mM phenylmethylsulfonyl fluoride. Total protein concentrations were measured using a bicinchoninic acid (BCA) protein assay kit (Beyotime Biotechnology, Nantong, China). Equal amounts of proteins were separated by 10% sodium dodecyl sulfate-polyacrylamide gel electrophoresis (SDS-PAGE). The proteins were then transferred onto polyvinylidene fluoride (PVDF) membranes, which were then blocked with skim milk (5%) in Tris-buffered saline containing 0.1% Tween-20 (Sigma-Aldrich) for 1 h at room temperature and incubated at 4°C overnight with primary antibodies, including anti-DDR1, anti-PI3K (anti-phosphatidylinositol 3 kinase), anti-p-AKT (anti-phosphorylation protein kinase B), anti-AKT (anti-protein kinase B), anti-MDM2 (anti-murine double minute 2), anti-P53, anti-PFKFB2 (anti-6-phosphofructo-2-kinase/fructose-2,6-bisphosphatase2), anti-PDHK1 (anti-pyruvate dehydrogenase kinase-1), anti-PKM2 (anti-M2 type pyruvate kinase), anti-Bcl2 (anti-B cell lymphoma/leukenfia 2), and anti-β-actin antibodies (1:1,000). Membranes were washed three times with Tris-buffered saline containing 0.05% Tween-20 (pH 7.2) and incubated with peroxidase-conjugated secondary antibodies (1:3,000) for 2 h at room temperature. Finally, blots were developed using ECL chemiluminescence reagents (Amersham Pharmacia Biotech, Cambridge, UK ). The protein band intensities were determined using Quantity One software (Bio-Rad Laboratories, Hercules, USA).

### Statistical analysis

All statistical analyses were performed using SPSS 22.0 (SPSS Inc, Chicago, USA). The *χ* ^2^ test or Fisher’s exact test was used to analyze the association between DDR1a expression and the clinicopathological parameters of the patients. Statistical comparisons of the results were evaluated using one-way ANOVA followed by Bonferroni’s multiple-comparison analysis. All experiments were independently repeated at least three times. Data are presented as the mean±standard deviation (SD), and statistical significance was set at *P*<0.05.

## Results

### DDR1 is correlated with the highly pathological T/N stage in CRC

**Figure 1:**
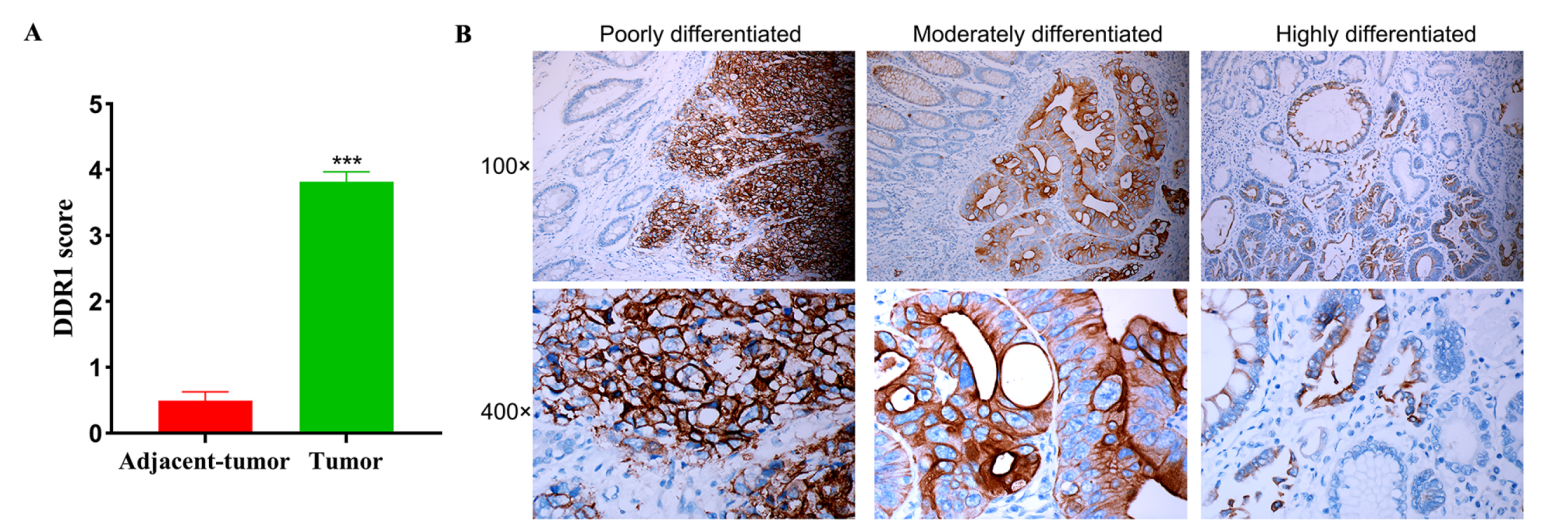
Figure1 The expression of DDR1 in CRC (A) The immunohistochemical score of DDR1 expression in the tumor and tumor-adjacent tissues. (B) Representative immunohistochemical images of DDR1 expression in tissues from patients with poorly-moderately-highly differentiated adenocarcinoma at ×100 and ×400 magnification. ***P<0.001.

We evaluated DDR1 expression in CRC tissues using immunohistochemical analysis. The specimens were collected from 126 patients (72 males and 54 females) with a median age of 61.33±10.93 years. There was a significant difference in DDR1 expression levels between CRC and adjacent normal tissues ( Figure 1A,B). According to the immunohistochemical scores listed in Table 2, the DDR1 positive rate was 100% (34 of 34) in poorly differentiated carcinoma tissues, 76% (57 of 75) in moderately differentiated, and 29.4% (5 of 17) in highly differentiated carcinoma tissues. The clinical physiological indices shown in Table 1 demonstrated that DDR1 expression was positively correlated with tumor size, pT/N stage, vascular invasion, and clinical AJCC stage. Collectively, these data confirmed that DDR1 is highly involved in CRC progression.

### DDR1a promotes LoVo cell proliferative and affects mitochondrial function

**Figure 2:**
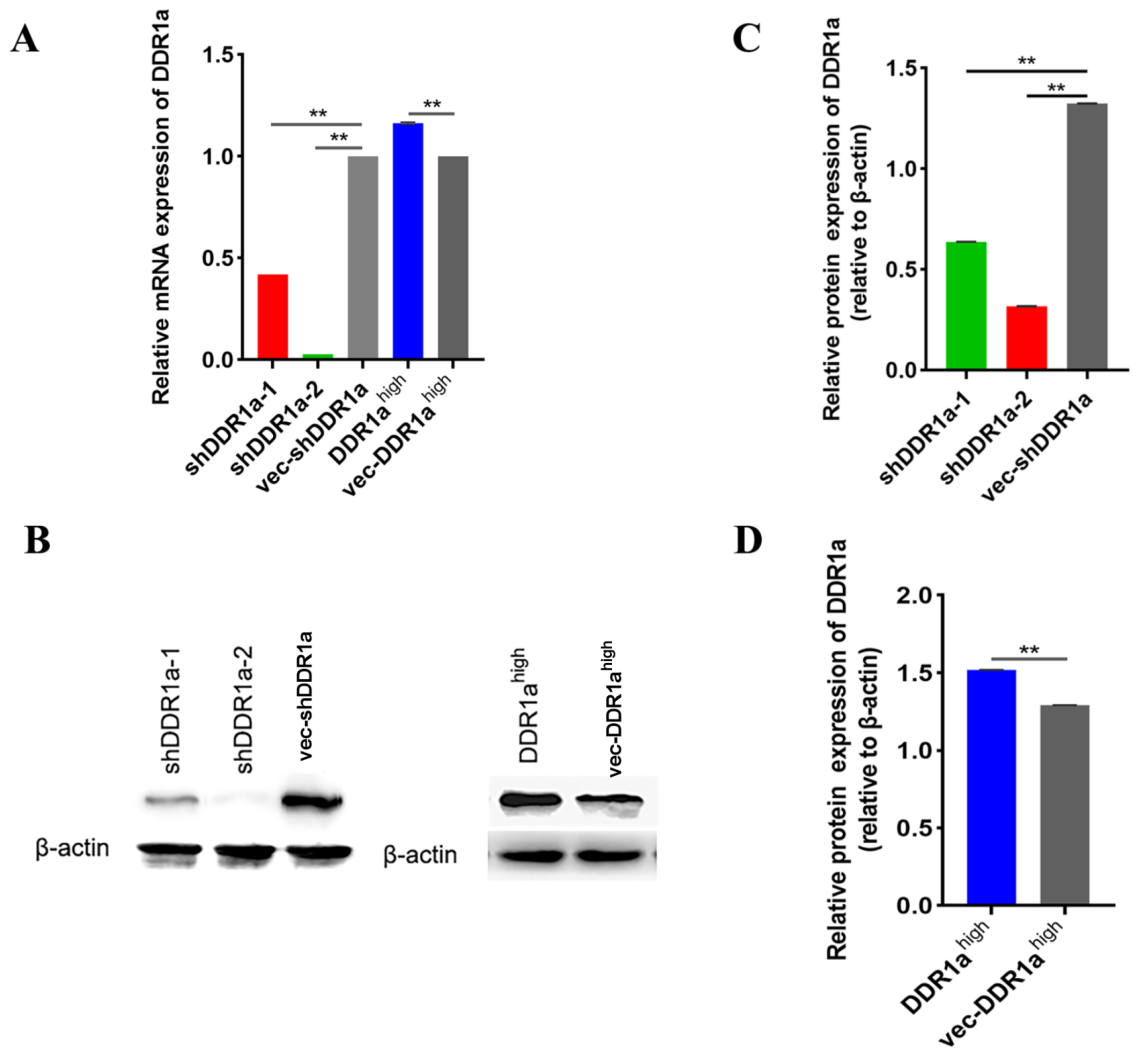
Figure2 The transfection efficiency of lentivirus vector-mediated knockdown and overexpression of DDR1 Two vectors with short hairpin RNAs targeting DDR1a (LV3-DDR1a-homo-1733 and LV3-DDR1a-homo-1043) and one vector with DDR1a overexpression were constructed and transfected into LoVo cells. The relative expression level of DDR1a was determined by qRT-PCR (A) and western blot analysis (B–D) 72 h post-transfection.**P<0.01. shDDR1a-1: LV3-DDR1a-homo-1733, shDDR1a-2: LV3-DDR1a-homo-1043, vec-shDDR1a: vector control, DDR1ahigh: DDR1a overexpression, vec-DDR1ahigh: vector control.

**Figure 3:**
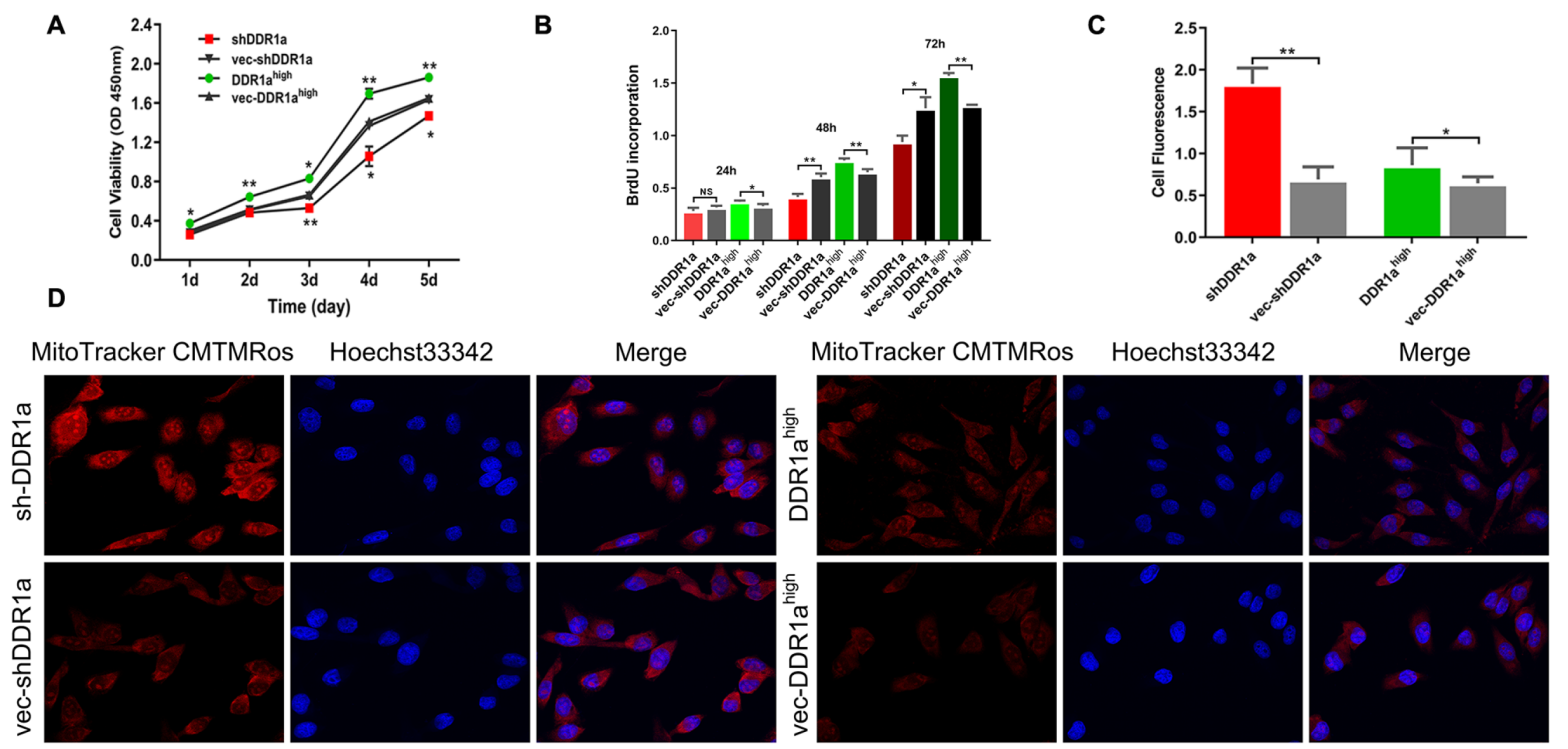
Figure3 DDR1a promotes the LoVo cell proliferation by regulating mitochondrial activity LoVo cells were transfected with DDR1a short hairpin RNA lentiviral vector, DDR1a overexpression lentiviral vector (DDR1ahigh) or their corresponding negative controls. (A,B) Cell proliferation was determined by CCK-8 (A) and BrdU assays (B). (C) MitoTracker Orange fluorescence intensity was quantified using ImageJ software. *P<0.05, **P<0.01. shDDR1a or DDR1ahigh vs negative control. NS, no significance. (D) Cells were incubated with MitoTracker Orange (20 nM) for 1 h. Hoechst 33342 (2 μg/mL) was used to stain the nuclei. Fluorescence images were captured at 200× magnification with a fluorescence microscope. shDDR1a: LV3-DDR1a-homo-1733, vec-shDDR1a: vector control, DDR1ahigh: DDR1a overexpression, vec-DDR1ahigh: vector control.

To determine the role of DDR1a in CRC, cells were transfected with sh-DDR1a or DDR1a overexpression vectors, and transfection efficiency was assessed via qPCR or western blot analysis ( Figure 2A–D). The observed cell viability indicated that LoVo cell proliferation was stimulated by DDR1a overexpression and inhibited by DDR1a knockdown ( Figure 3A). The BrdU assay confirmed that overexpression of DDR1a promoted LoVo cell proliferation significantly, especially after 48 h compared with DDR1a knockdown ( Figure 3B). MitoTracker Orange fluorescence staining indicated a significant increase in fluorescence intensity in DDR1a-knockdown cells. The mean fluorescence intensity of DDR1a-knockdown cells was 3-fold stronger than that of the control ( Figure 3C,D). Therefore, we preliminarily concluded that DDR1a directly affects mitochondrial function.

### DDR1a inhibition affects energy supply from aerobic oxidation for cell proliferation

**Figure 4:**
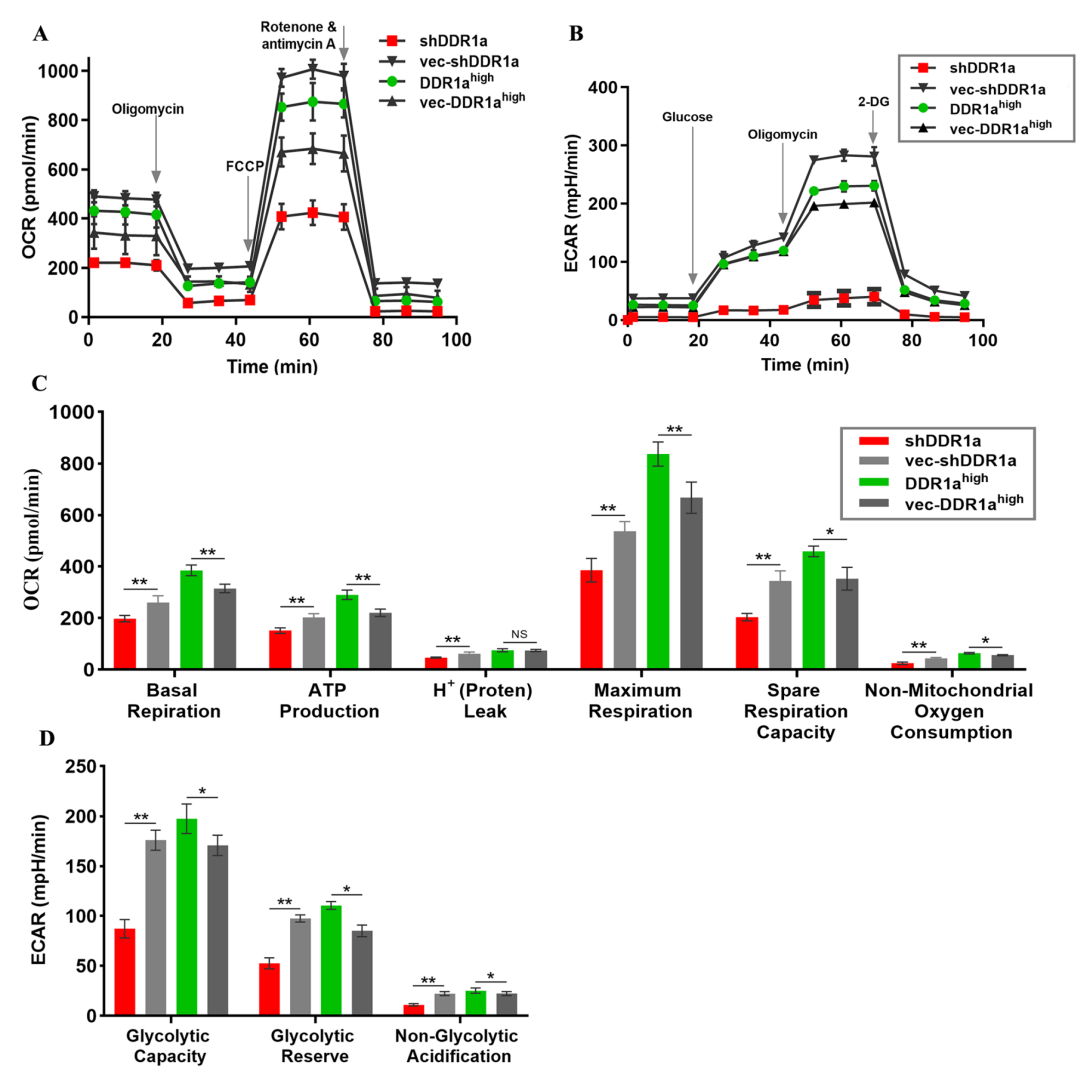
Figure4 DDR1a reprograms the energy metabolism of LoVo cells (A) Oxygen consumption rate (OCR). (B) Extracellular acidification rate (ECAR). There are no significant differences among the groups (Kruskal–Wallis nonparametric test; n=3–4). (C) Parameters for mitochondrial respiration. (D) Parameters for glycolysis. 2-DG: 2-Deoxyglucose, FCCP: Carbonyl cyanide p-(tri-fluromethoxy)phenyl-hydrazone], shDDR1a: LV3-DDR1a-homo-1733, vec-shDDR1a: vector control, DDR1ahigh: DDR1a overexpression, vec-DDR1ahigh: vector control. *P<0.05, **P<0.01.

The Seahorse XF-24 Extracellular Flux Analyzer was applied in the experiments to further evaluate the effect of DDR1a on mitochondrial glycolytic capacity in real-time. To investigate whether the respiratory chain enzyme complexes in mitochondria are also affected, OCR was measured after sequentially adding oligomycin (to inhibit ATP synthase), FCCP (to uncouple the mitochondrial inner membrane and allow for maximum electron flux through the ETC), rotenone (to inhibit complex I), and antimycin A (to inhibit complex III). The differences in basal respiration, ATP production, H ^+^ (proton) leak, maximum respiration, spare respiration capacity, and non-mitochondrial oxygen consumption confirmed weakened mitochondrial function in shDDR1a-transfected cells ( Figure 4A,C).

### DDR1a inhibition upsets acid-base microenvironment balance

**Figure 5:**
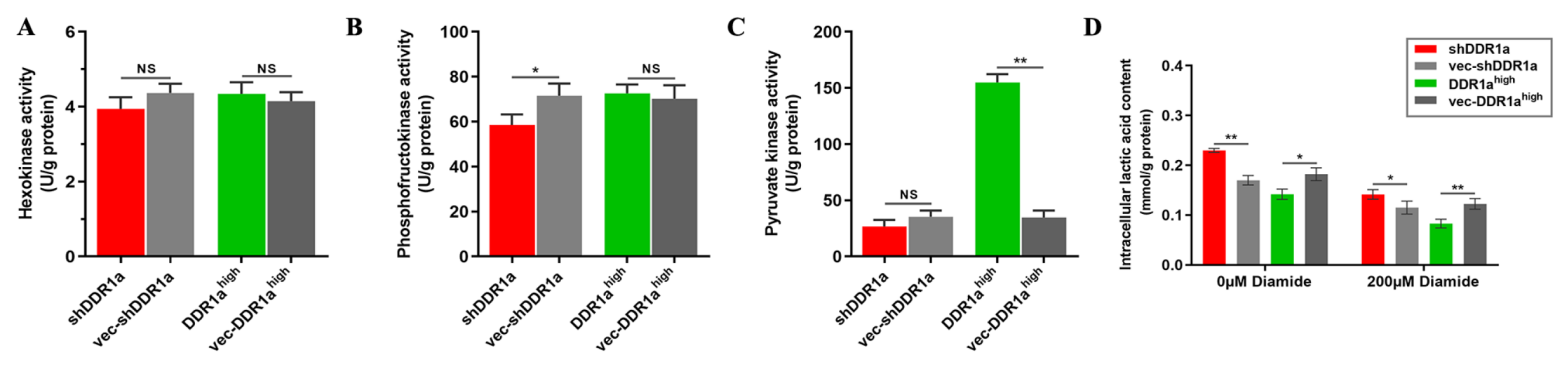
Figure5 Inhibition of DDR1a exacerbates intracellular retention of lactate (A–C) Hexokinase (HK), phosphofructokinase (PFK), and pyruvate kinase (PK) activities were determined by UV spectrophotometry. (D) The intracellular lactate content in the control and 200 μM diamide treatments. shDDR1a: LV3-DDR1a-homo-1733, vec-shDDR1a: vector control, DDR1ahigh: DDR1a overexpression, vec-DDR1ahigh: vector control. *P<0.05, **P<0.01.

As shown in Figure 4B, the ECAR was elevated in DDR1a-overexpressing cells compared to the shDDR1a cells. After sequential addition of saturating concentrations of glucose, oligomycin, and 2-deoxy-glucose (2-DG), the rate of glycolysis under basal conditions, glycolytic capacity, and glycolytic reserve were determined. The glycolytic capacity, glycolytic reserve, and non-glycolytic acidification in shDDR1a cells were reduced ( Figure 4D). At the same time, the intracellular lactate content was examined. Significantly increased intracellular lactate content in shDDR1a cells ( Figure 5D) implies that the retention of large amounts of lactic acid induces intra- and extracellular microenvironment disturbances.

### DDR1a regulates intra- and extracellular lactic acid content by activating PK

HK, PFK, and PK are three key kinases involved in aerobic glycolysis. In the present study, only PK activity was found to be apparently upregulated by DDR1a overexpression, whereas HK and PFK were unaffected ( Figure 5A–C). The intracellular lactic acid content in DDR1a-overexpressing and DDR1a-knockdown cells were both decreased when cells were cultured in medium containing diamide (200 μM), a pyruvate kinase inhibitor. However, the lactic acid content in DDR1a-knockdown cells was still higher than that in the control ( Figure 5D), suggesting that DDR1a could upregulate PK activity, and the PK inhibitor could not reverse intracellular lactic acid retention caused by DDR1a inhibition.

### DDR1a regulates pyruvate kinase via PI3K/AKT/PKM2 signaling pathway

**Figure 6:**
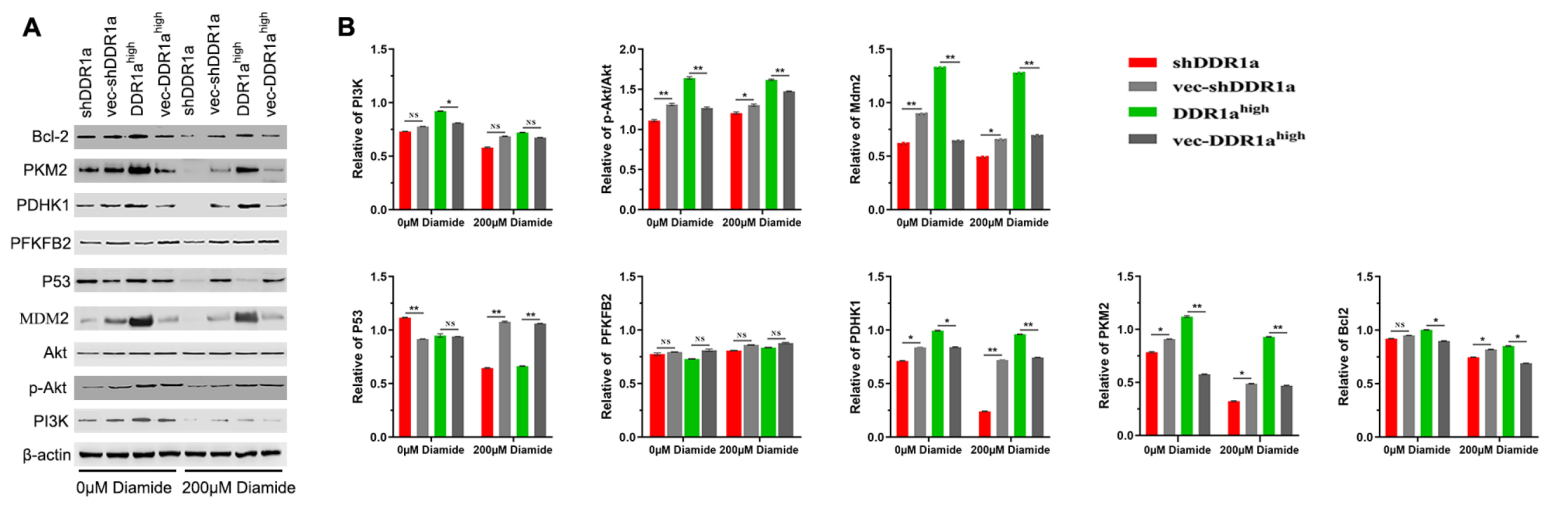
Figure6 DDR1a promotes LoVo cell proliferation by the PI3K/AKT/PKM2 pathway (A) Representative western blots of the expressions of selected proteins. (B) Protein expression levels of PI3K, p-AKT, and MDM2 were determined by western blot analysis. β-Actin was used as the loading control. *P<0.05, **P<0.01 for shDDR1a or DDR1ahigh vs their corresponding negative controls. shDDR1a: LV3-DDR1a-homo-1733, vec-shDDR1a: vector control, DDR1ahigh: DDR1a overexpression, vec-DDR1ahigh: vector control.

Previous studies have revealed that the PI3K/AKT/mTOR signaling pathway plays a crucial role in regulating cell growth, survival, and metabolism [28]. In this study, western blot analysis results revealed that DDR1a overexpression promoted the expressions of PI3K, p-AKT, MDM2, PDHK1, PKM2, and Bcl-2 proteins, whereas DDR1a knockdown inhibited the expressions of these proteins. However, there was no statistically significant differential expression of p53 between the DDR1a-overexpressing cells and the negative control cells, while DDR1a knockdown promoted p53 expression ( Figure 6A). In addition, treatment with diamide (200 μM) for 15 min decreased the p-AKT level, as well as the PI3K, MDM2, PDHK1, PKM2 and Bcl2 levels in both the DDR1-overexpressing cells and DDR1-knockdown cells ( Figure 6B). These findings indicate that the PI3K/AKT/PKM2 signaling pathway bridges glucose metabolism and cell growth in LoVo cells.

## Discussion

DDR1 is characterized by its collagen-bound external dish protein homeodomain, which is a central extracellular matrix sensor that regulates cell adhesion, proliferation, motility, and invasion [ 17, 26, 29]. In the present study, through histopathological analysis, we demonstrated that DDR1 was highly expressed in CRC tissues. Moreover, a relatively higher DDR1 expression level in patients correlated with a highly advanced TNM stage and poor differentiation. *In vitro*, we found that LoVo cell proliferation was stimulated and depressed by DDR1a overexpression and inhibition, respectively. These results prompted us to further investigate the role of DDR1 in tumor initiation and progression.

Rewiring cellular metabolism as an adaptive regulator of the microenvironment provides cancer cells with energy [30] and biosynthetic precursors [ 31, 32]. This mechanism has been reported to be involved in the pathogenesis of CRC. Different cell viability states were observed in shDDR1a-transfected and DDR1a-overexpressing cells, and mitochondrial fluorescence was enhanced in both groups *in vitro* . It is not known how DDR1a influences the mitochondrial membrane potential by altering the LoVo cell glycolysis, and further affects tumorigenesis. Our data showed that ECAR in the DDR1a-overexpressing cells was higher than that in the control cells. Similar to most tumor cells, DDR1a-overexpressing cells rely on the “Warburg effect” to gain a survival or growth advantage. Although mitochondrial fluorescence intensity was 3-fold higher in shDDR1a-transfected cells than in the control cells, the OCR and ECAR values in shDDR1a-transfected cells were even lower than those in the control cells with ATP synthase inhibitor or uncoupling agent. Evidently, once oxidative phosphorylation is impaired, excessive lactic acid and ATP would lead to mitochondrial membrane potential dysfunction and even cause increased proton leakage. Thus, we speculate that DDR1a inhibition may curb the aggressive behavior of LoVo cells by metabolic reprograming.

It is well known that lactic acid is a metabolic by-product, and the acid-base imbalance microenvironment directly affects cell mitosis and proliferation. In shDDR1a-transfected cells, we observed a downward trend in ECAR value, but surprisingly, we also observed a higher intracellular lactic acid content in the shDDR1a-transfected cells than in the control cells. Because of DDR1a inhibition, a large amount of lactic acid from the fermentation pathway accumulates within the cell, and rapidly induces the microenvironment changes; therefore, the Warburg effect, which compensates for energy metabolism, is lost in LoVo cells. Consequently, mitochondrial depolarization induced by FCCP and ATP synthase inhibitors provides intracellular carbon sources, an “invalid loop,” resulting in shDDR1-transfected cells becoming overwhelmed. Therefore, our results are consistent with previous studies which reported that the distinctly different methods by which tumor cells utilize carbon fuel contribute to their invasive behavior [ 33– 35] and tumor pathogenesis [ 36, 37].

It is unclear which enzyme is key to glycolysis regulation in LoVo cells. Therefore, the enzymatic activities of HK, PFK, and PK were determined in the present study. Compared with the control, PK activity was apparently upregulated by DDR1a overexpression, while HK and PFK were unaffected. Diamide, a small-molecule PK inhibitor, significantly reduced the lactate content in DDR1a-knockdown cells, exacerbating intracellular acidosis caused by lactic acid accumulation. This result suggests that PK inhibitors might be effective synergists if DDR1a could be used as a therapeutic target for CRC.

Many signaling pathways can regulate metabolism and are associated with cell metabolism [ 38, 39]. The RTK/PI3K/AKT/mTOR signaling pathway plays a crucial role in regulating cell growth, survival, and metabolism [ 40, 41]. PKM2 activates PI3K/Akt and then stimulates Wnt/β-catenin signaling to promote cell migration of colon cancer cells [42]. Western blot analysis showed that DDR1a overexpression promoted the expression levels of PI3K, p-AKT, MDM2, PDHK1, and PKM2 proteins, although there was no significant difference in total AKT and PFKFB2 expression between DDR1a-knockdown and DDR1a-overexpressing cells and their corresponding controls. In addition, treatment with 200 μM diamide for 15 min decreased the p-AKT level in both DDR1a-overexpressing and DDR1a-knockdown cells, as well as the PI3K, MDM2, PDHK1, PKM2, and Bcl2 levels. Our results demonstrate that the PI3K/AKT/PKM2 signaling pathway is associated with glucose metabolism in LoVo cells.

**Figure 7:**
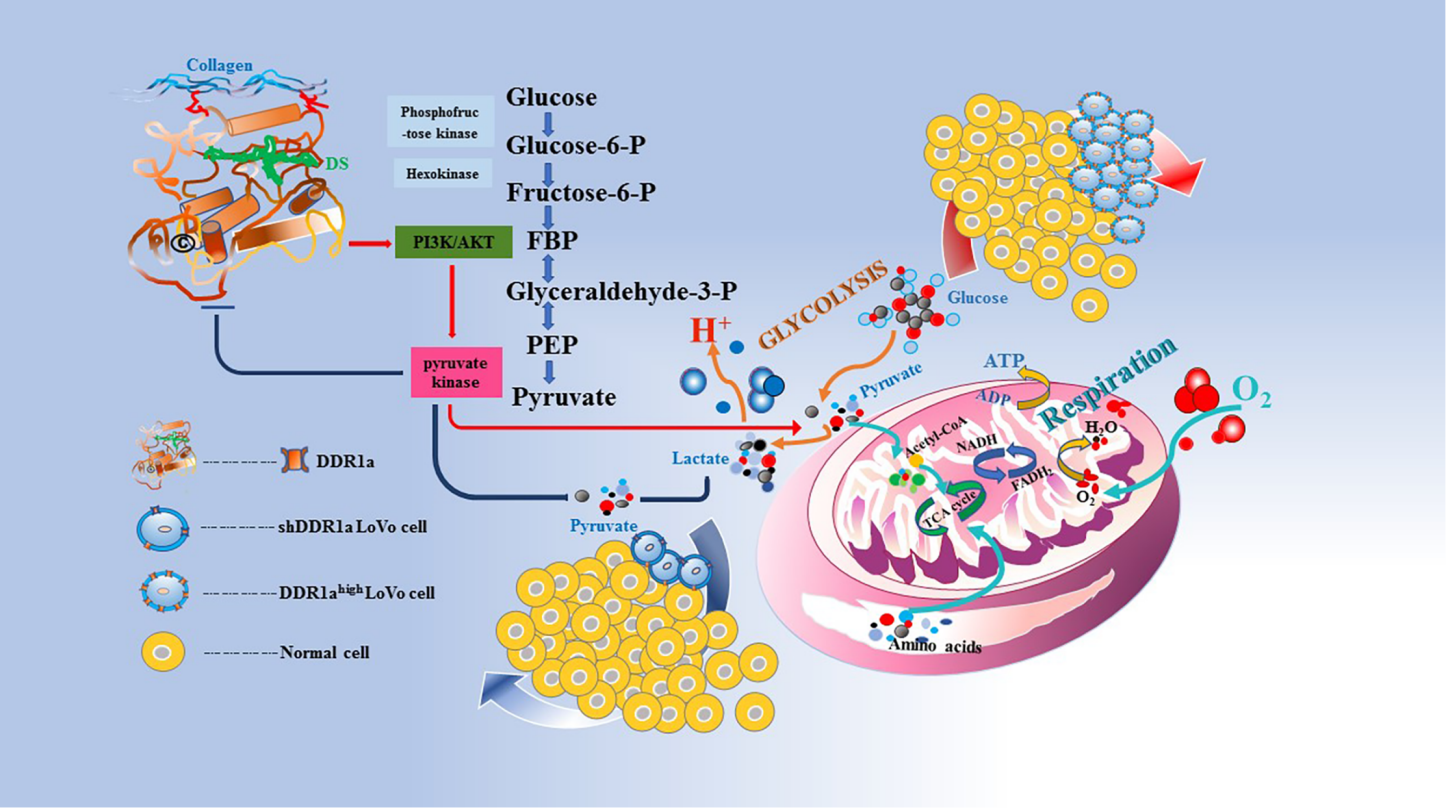
Figure7 Schematic diagram of the role of DDR1a in LoVo cell metabolic programing DDR1, as a centeral extracellular matrix sensor, is involved in cell adhesion, proliferation and invasion. DDR1a inhibition downregulates pyruvate kinase activity via PI3K/AKT/PKM2 signaling pathway, promoting intracellular lactate accumulation and metabolic reprogramming in LoVo cells.

In summary, DDR1 may be a critical factor involved in LoVo cell proliferation by regulating intracellular and extracellular lactic acid content. This can be achieved by activating pyruvate kinase and reprogramming CRC cell metabolism by mediating the PI3K/AKT/PKM2 signaling pathway. The mechanism is illustrated by a diagram shown in Figure 7. Nevertheless, the present study has some limitations. We used only one cell line (LoVo cells) and one inhibitor (diamide) in our experiments. Further studies are needed in the future to fully uncover the functions and underlying mechanisms of DDR1 in cell energy metabolism
